# Cognitive impairment and dementia in a nationally representative sample of older adults receiving care for incident TBI

**DOI:** 10.1002/alz.086561

**Published:** 2025-01-09

**Authors:** Erica S Kornblith, L. Grisell Diaz‐Ramirez, Kristine Yaffe, W John Boscardin, Raquel C. Gardner

**Affiliations:** ^1^ San Francisco Veterans Affairs Health Care System, San Francisco, CA USA; ^2^ University of California, San Francisco, San Francisco, CA USA; ^3^ University of California San Francisco / San Francisco VA Medical Center, San Francisco, CA USA; ^4^ NCIRE‐The Veterans Health Research Institute, San Francisco, CA USA; ^5^ Sheba Medical Center, Joseph Sagol Neuroscience Center, Ramat Gan Israel

## Abstract

**Background:**

Cognitive problems are thought to increase vulnerability to geriatric traumatic brain injury (TBI) due to increased fall risk, but little is known about prevalence of cognitive impairment and Alzheimer’s disease and related dementias (ADRD) among elders who receive treatment for a TBI.

**Method:**

Enrollees 65 and older in the nationally representative Health and Retirement Study (HRS) who consented to link survey data to Medicare claims and without a TBI prior to enrollment were studied. We used claims 2000‐2018 to obtain incident TBI diagnoses, defined using inpatient and outpatient International Classification of Disease (ICD) 9 and 10 codes received the same day as an emergency room (ER) visit code and a computed tomography (CT) scan code. Sociodemographic and health factors were defined at baseline interview. Baseline cognition was defined using Langa‐Weir Dementia Probability, a predictive model that classifies each respondent as ADRD; cognitive impairment, no dementia (CIND); or normal cognition.

**Result:**

Of respondents who experienced incident TBI (n = 797), 74% were classified as normal cognition, 19% as CIND and 7.1% ADRD at baseline. In the no‐TBI group (n = 8442), 68% had normal cognition, 23% CIND, and 10% ADRD (p<0.01). In sensitivity analyses considering TBI ± ER treatment and/or CT, results were similar. However, in multivariate models examining association of incident TBI with baseline sociodemographic, medical, and cognitive characteristics, neither CIND nor ADRD were associated with TBI status (p’s = 0.30 and 0.51, respectively). Instead, Black race (p<0.01), male sex (p = 0.02), higher education (p = 0,01), lung disease (p<0.01), higher neighborhood socioeconomic status (SES, p<0.01), and older age (p<0.01) were associated with membership in the no‐TBI group.

**Conclusion:**

Our results suggest prevalence of baseline cognitive impairment and dementia is lower among elders treated for an incident TBI during study follow‐up, compared to the no‐TBI group. CIND/ADRD may protect against TBI through reduced activity or increased support with daily tasks. Alternatively, elders with CIND/ADRD may have reduced health care access or utilization. Moreover, demographic and contextual factors like age, gender, education, and neighborhood SES appear to be more meaningful predictors of incident TBI in this sample, vs. baseline cognition.

